# Supplementary material for: Association of SNPs in the PAI1 Gene with Disease Recurrence and Clinical Outcome in Bladder Cancer
Source: Int J Mol Sci. 2023 Mar 3;24(5):4943. doi: 10.3390/ijms24054943 (PMC10003630; doi:10.3390/ijms24054943)
Supplement: Supplementary file 1 [file ijms-24-04943-s001.zip › Supplemental Figures.pptx]

## Slide 1
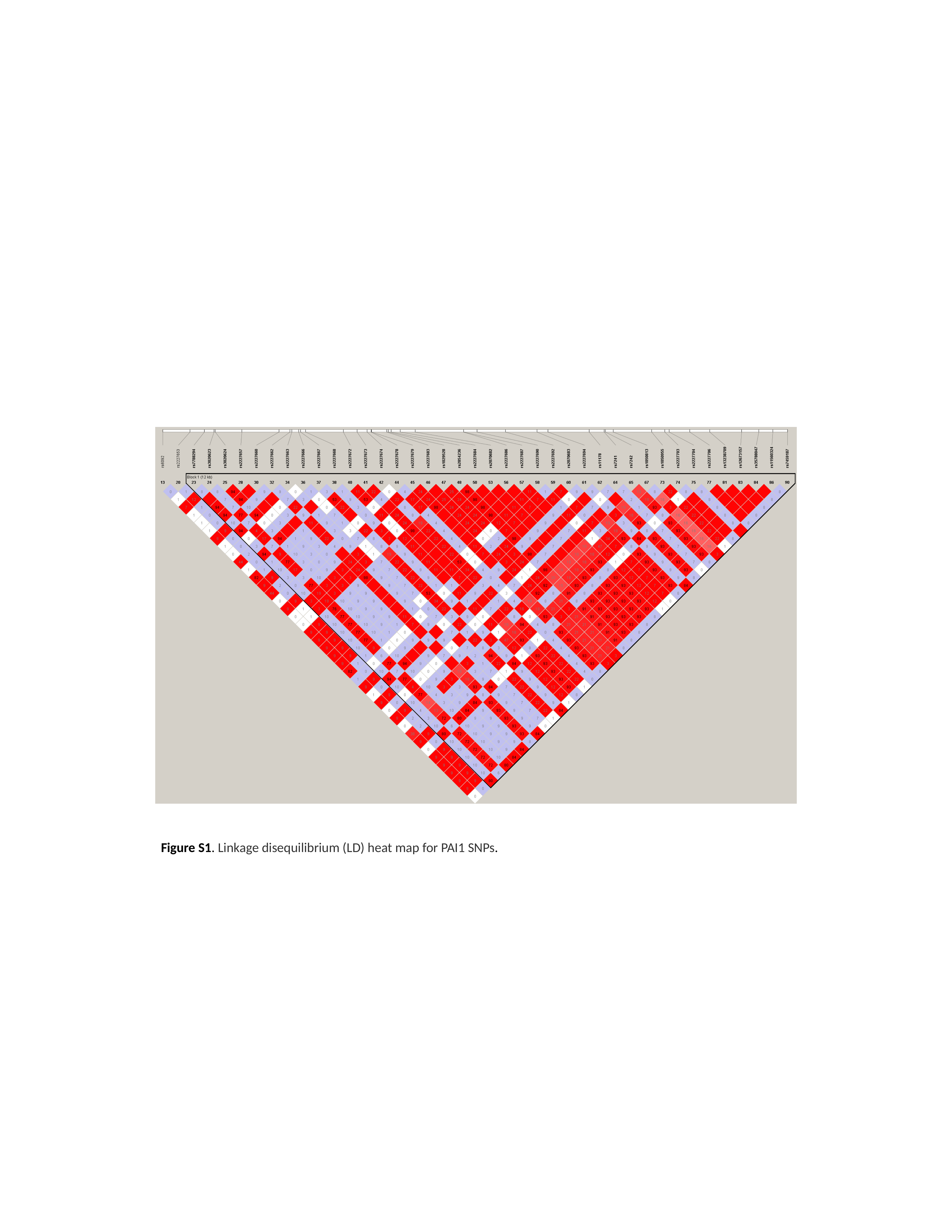

Figure S1. Linkage disequilibrium (LD) heat map for PAI1 SNPs.

## Slide 2
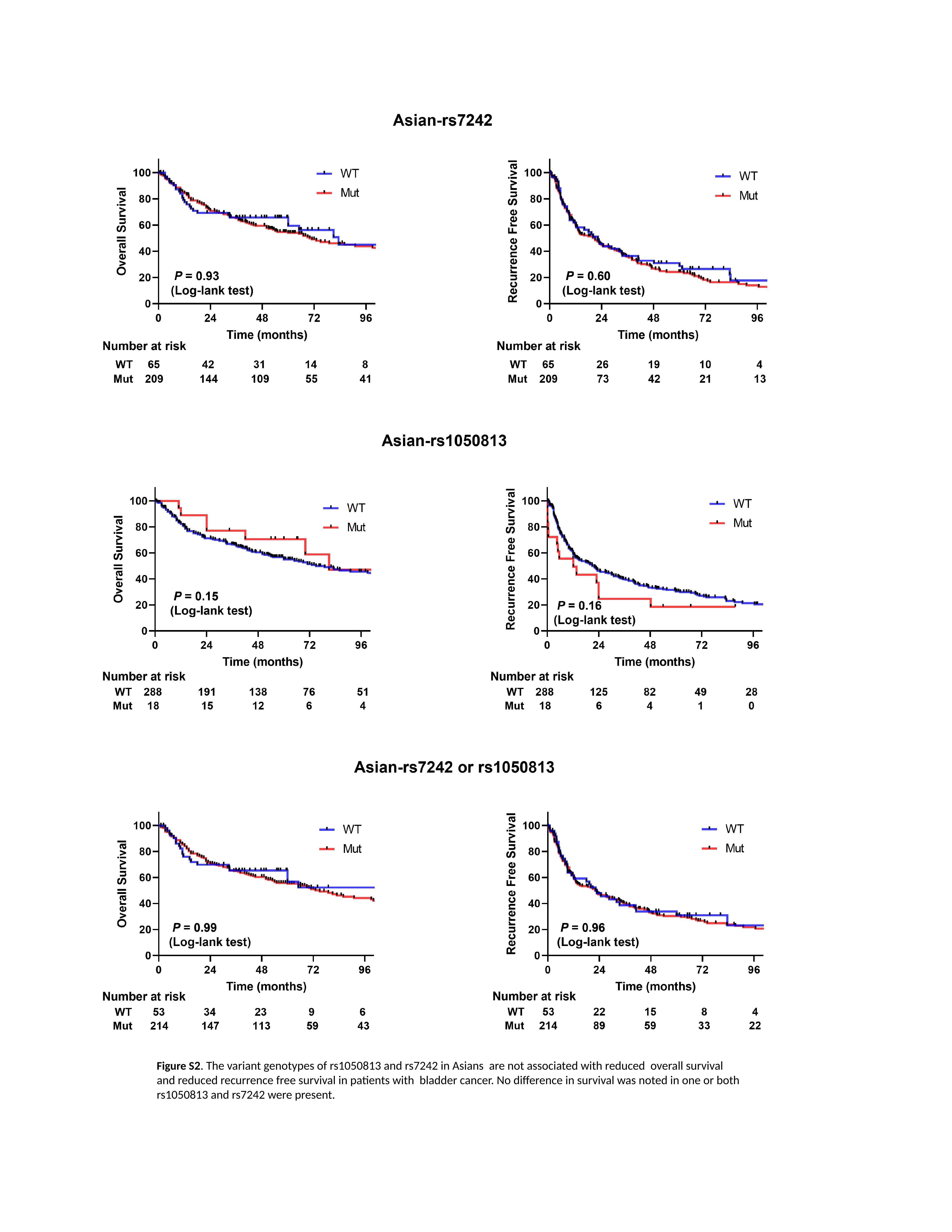

Figure S2. The variant genotypes of rs1050813 and rs7242 in Asians are not associated with reduced overall survival
and reduced recurrence free survival in patients with bladder cancer. No difference in survival was noted in one or both
rs1050813 and rs7242 were present.

## Slide 3
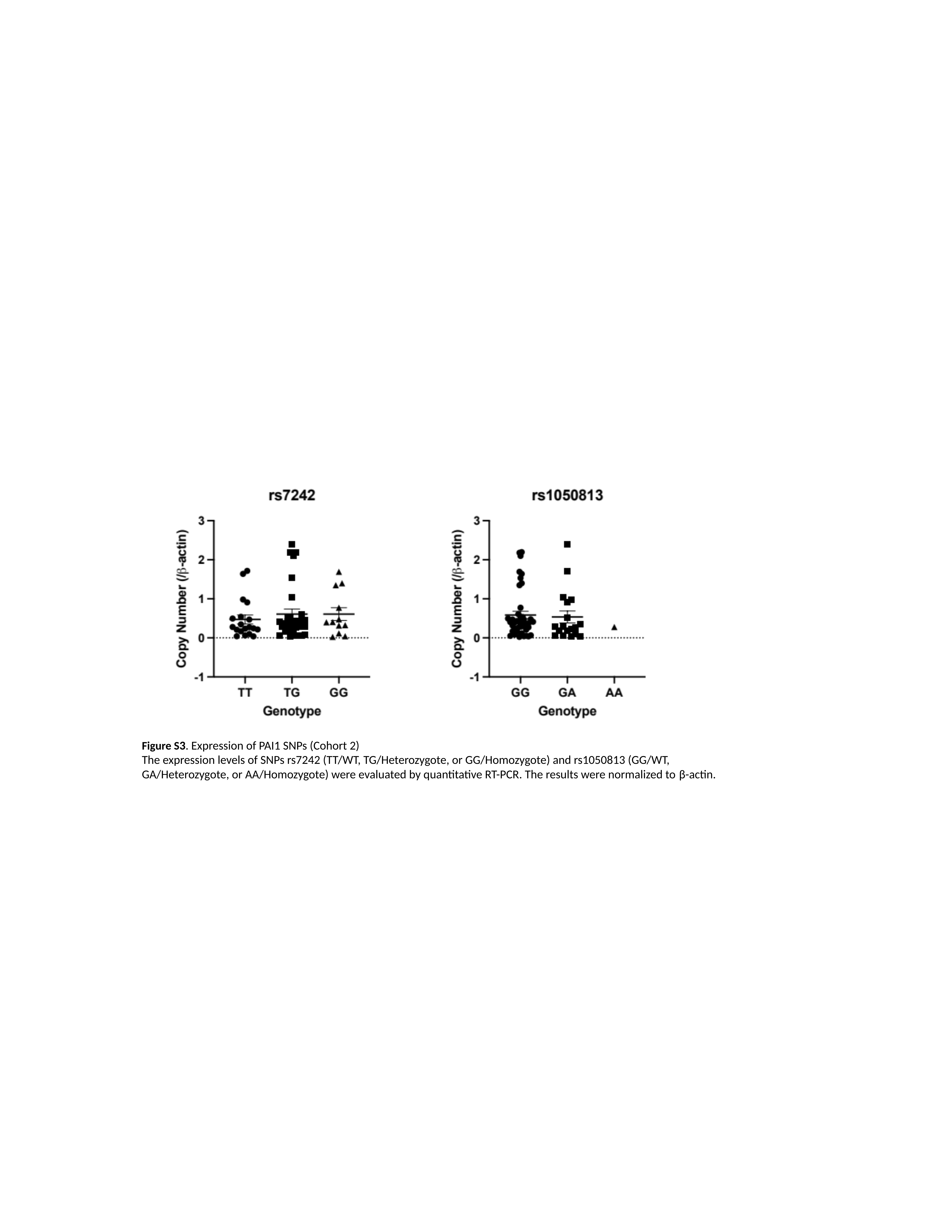

Figure S3. Expression of PAI1 SNPs (Cohort 2)
The expression levels of SNPs rs7242 (TT/WT, TG/Heterozygote, or GG/Homozygote) and rs1050813 (GG/WT, GA/Heterozygote, or AA/Homozygote) were evaluated by quantitative RT-PCR. The results were normalized to β-actin.

## Slide 4
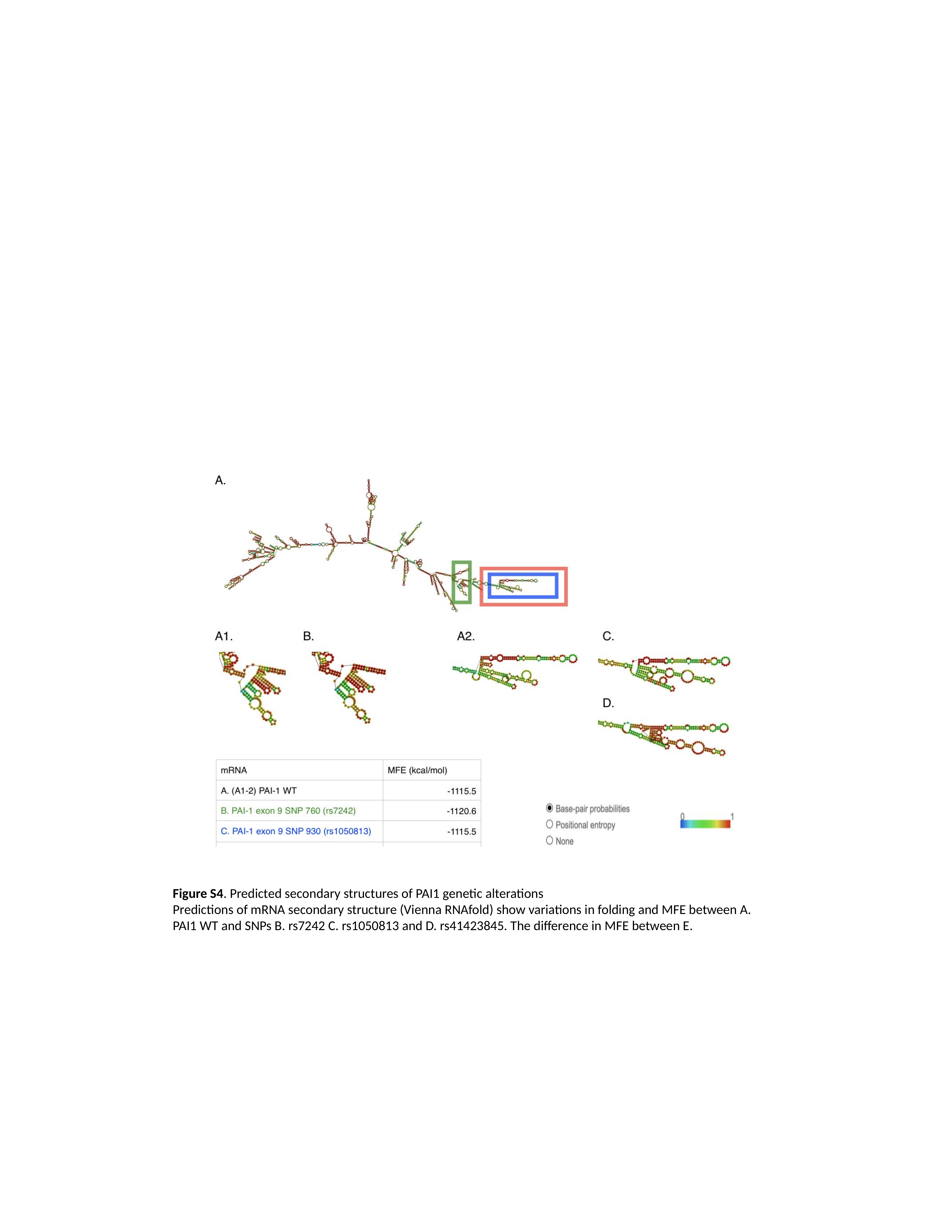

Figure S4. Predicted secondary structures of PAI1 genetic alterations
Predictions of mRNA secondary structure (Vienna RNAfold) show variations in folding and MFE between A. PAI1 WT and SNPs B. rs7242 C. rs1050813 and D. rs41423845. The difference in MFE between E.

## Slide 5
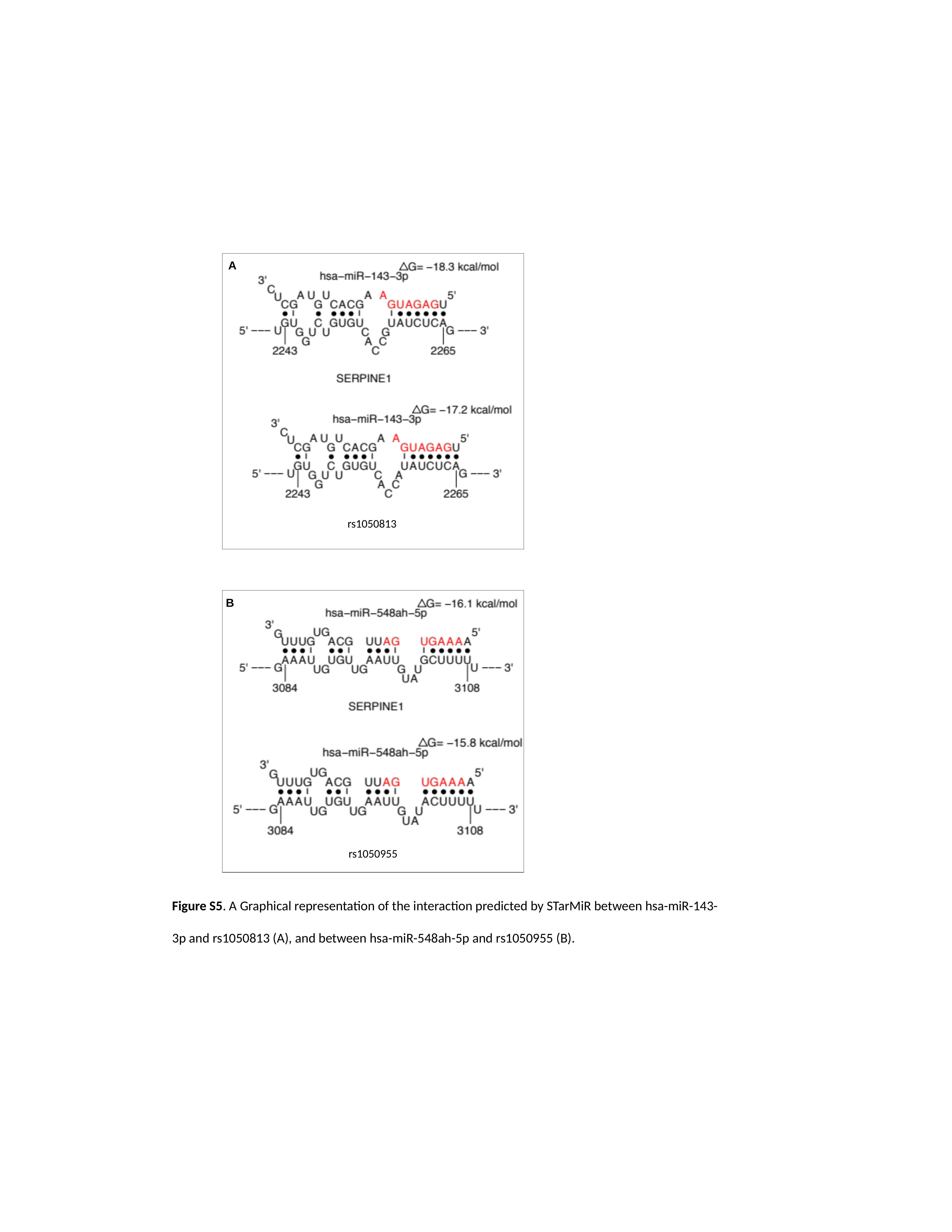

A
rs1050813
B
rs1050955
Figure S5. A Graphical representation of the interaction predicted by STarMiR between hsa-miR-143-3p and rs1050813 (A), and between hsa-miR-548ah-5p and rs1050955 (B).

## Slide 6
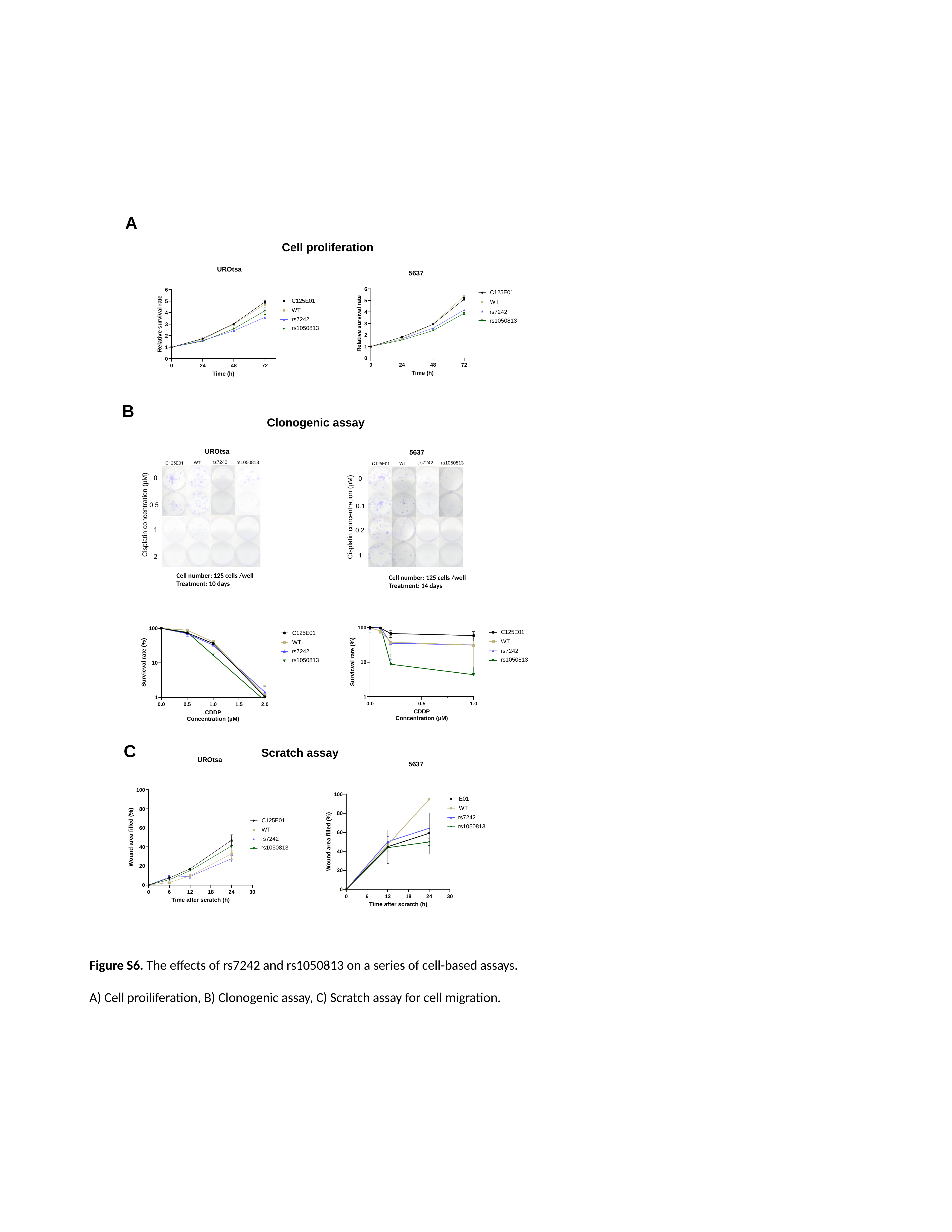

A
Cell proliferation
UROtsa
5637
rs7242
rs7242
rs1050813
rs1050813
B
Clonogenic assay
UROtsa
5637
Cisplatin concentration (µM)
Cisplatin concentration (µM)
Cell number: 125 cells /well
Treatment: 10 days
Cell number: 125 cells /well
Treatment: 14 days
rs7242
rs1050813
rs7242
rs1050813
rs7242
rs7242
rs1050813
rs1050813
C
Scratch assay
UROtsa
5637
rs7242
rs1050813
rs7242
rs1050813
Figure S6. The effects of rs7242 and rs1050813 on a series of cell-based assays.
A) Cell proiliferation, B) Clonogenic assay, C) Scratch assay for cell migration.
