# Supplementary material for: Association of SNPs in the PAI1 Gene with Disease Recurrence and Clinical Outcome in Bladder Cancer
Source: Int J Mol Sci. 2023 Mar 3;24(5):4943. doi: 10.3390/ijms24054943 (PMC10003630; doi:10.3390/ijms24054943)
Supplement: Supplementary file 1 [file ijms-24-04943-s001.zip › Supplementary methods.pdf]

SUPPLEMENTAL MATERIAL

RT-PCR Primers

To perform the amplification of *PAI1 transcripts* the following primers were used; *PAI1* 1 (exons 1-6) forward 5’-CAAGAAGACCCACACGCCC-3’, reverse 5’- CGGTCATTCCCAGGTTCTCT-3’; *PAI1* 2 (exons 5-9) forward 5’-ACTACGACATCCTGGAACTGC-3’, reverse 5’-AGTGGACTCTGAGATGAAAGGG-3’; *PAI1* 3 (exon 9) forward 5’-TGGAACTACGGGGCTTACAG-3’, reverse 5’-ATCCATCTTTGTGCCCTACCC-3’.

PAI1, forward primer: 5′–GCC AGT GGA AGA CTC CCT TC –3′, reverse primer: 5′– GTG CTG CCG TCT GAT TTG TG –3′, *CLSPN*, forward primer: 5′–CTG GGC TAT TTC GAG CCA GT –3′, reverse primer: 5′– GCT CTG GGG AGG CGT TAT AC –3′, *ATR*, forward primer: 5′– TGG GAC CCA AAC ATG TCA GT –3′, reverse primer: 5′– ATG ATC CAG GCA GCG AAC AA –3′; *CHEK1*, forward primer: 5′– CTC CTC TAG CTC TGC TGC AT –3′, reverse primer: 5′– AAG TGA CTC GGG GCC TTT TT –3′; and β-actin, forward primer: 5′–TCC TCC CTG GAG AAG AGC TA–3′, reverse primer: 5′–CCA GAC AGC ACT GTG TTG GC–3′.

mRNA expression analysis

cDNA was subjected to real-time PCR amplification of *PAI1* for 40 cycles. The following primers were used for real-time PCR amplification: *PAI1*, forward 5'-GCCAGTGGAAGACTCCCTT-3' and reverse 5'-GTGCTGCCGTCTGATTTGTG-3'. β-actin was used to estimate the efficiency of cDNA synthesis. For alternative splicing detection, PCR and agarose gel electrophoresis were employed. RNA from Cohort 3 was reverse transcribed to single strand cDNA using High Capacity cDNA Reverse Transcription Kit (Applied Biosystems, Grand Island, NY, USA). To perform the amplification of transcripts occurring in *PAI1* the following primers were used: *PAI1* pair 1 (exons 1-6), forward 5'-CAAGAAGACCCACACGCCC-3' and reverse 5'-CGGTCATTCCCAGGTTCTCT-3'; *PAI1* pair 2, (exons 5-9) forward 5'-ACTACGACATCCTGGAACTGC-3' and reverse 5'-AGTGGACTCTGAGATGAAAGGG-3'; *PAI1* pair 3 (exon 9), forward 5'-TGGAACTACGGGGCTTACAG-3' and reverse 5'-ATCCATCTTTGTGCCCTACCC-3'. PCR product sizes were visualized on a 1.5% agarose gel.

Overexpression of PAI1 SNPs in UROtsa and 5637 cell lines

pDONR221 plasmids harboring the full length of PAI1 wild type (WT), rs7242 or rs1050813 were produced by GenScript (Piscataway, NJ). pDONR221 plasmids

were then subcloned into pDEST26 (Thermo Fisher Scientific, Waltham, MA). Transfection of plasmids was performed using jetPRIME (Polyplus, Vectura, France) according to the manufacturer's instruction. Briefly, cells were seeded at 1.5 x 10^5 cells per well of a 6-well plate in 2 ml of RPMI for 5637 or low glucose DMEM for UROtsa containing 10% FBS, 1% Penicillin-Streptomycin and incubated at 37°C and 5% CO_2 for 24 h. PAI1-WT-pDEST26, PAI1- rs7242-pDEST26, or PAI1-rs1050813-pDEST26 were used as plasmid DNA. C125E01 in pDONR223 (Kind gift from Dr. Dominic Esposito, NIH/NCI) was also subcloned into pDEST26 (C125E01-pDEST26) and this plasmid was used as a negative control. Two µg of plasmid DNA was used for plasmid transfection using jetPRIME. Transfection media were replaced after 4 h by cell growth medium. Stable transfectants were selected with 2000 mg/ml of G418 (Thermo Fisher Scientific) for 14 days and subcloned by limiting dilution in 96-well plates. Stable cell lines were maintained in media containing 500 mg/ml of G418.

Labeling and purification

Microarray assay was performed by Psomagen. Total RNA extracted from cells using RNeasy Plus Mini Kit (Qiagen) was amplified and purified using

TargetAmp-Nano Labeling Kit for Illumina Expression BeadChip (EPICENTRE, Madison, USA) to yield biotinylated cRNA according to the manufacturer's instructions. Briefly, 250 ng of total RNA was reverse transcribed to cDNA using a T7 oligo(dT) primer. Second-strand cDNA was synthesized, *in vitro* transcribed, and labeled with biotin-NTP. After purification, the cRNA was quantified using the ND-1000 Spectrophotometer (NanoDrop, Wilmington, USA).

Hybridization and data export

750 ng of labeled cRNA samples were hybridized to each Human HT-12 v4.0 Expression Beadchip for 17h at 58°C, according to the manufacturer's instructions (Illumina, Inc., San Diego, USA). Detection of array signal was carried out using Amersham fluorolink streptavidin-Cy3 (GE Healthcare Biosciences, Little Chalfont, UK) following the bead array manual. Arrays were scanned with an Illumina bead array reader confocal scanner according to the manufacturer's instructions.

Raw data preparation and data analysis

The quality of hybridization and overall chip performance were monitored by visual inspection of both internal quality control checks and the raw scanned data. Raw data were extracted using the software provided by the manufacturer (Illumina GenomeStudio v2011.1 (Gene Expression Module v1.9.0)). Array probes transformed by logarithm and normalized by quantile method. Statistical significance of the expression data was determined using LPE test and fold change in which the null hypothesis was that no difference exists among groups. False discovery rate (FDR) was controlled by adjusting *p* value using Benjamini-Hochberg algorithm. For a DEG set, Hierarchical cluster analysis was performed using complete linkage and Euclidean distance as a measure of similarity. Pathway analysis was performed using Panther tools (http://www.pantherdb.org/tools/).

mRNA expression of PAI1, CLSPN, ATR, and CHEK1 genes

Total RNA was extracted from stable transfectants of UROtsa and 5637 cells using RNeasy Mini Kit (Qiagen) according to the manufacturer's protocols, and complementary DNA was synthesized from 1 µg of RNA using the ReverTra Ace qPCR RT Kit (TOYOBO, Osaka, Japan). We performed qRT-PCR using

SYBR Green Master Mix (Applied Biosystems, Foster City, CA, USA) and QuantStudio Real-time PCR system (Applied Biosystems). The thermal cycling conditions were 95°C for 15 s, 58°C for 60 s. The copy numbers of mRNA were determined after normalization to β-actin by using absolute quantification. All reactions were performed in triplicate. The primer sequences were as follows: *PAI1*, forward primer: 5′–GCC AGT GGA AGA CTC CCT TC –3′, reverse primer: 5′– GTG CTG CCG TCT GAT TTG TG –3′, *CLSPN*, forward primer: 5′–CTG GGC TAT TTC GAG CCA GT –3′, reverse primer: 5′– GCT CTG GGG AGG CGT TAT AC –3′, *ATR*, forward primer: 5′– TGG GAC CCA AAC ATG TCA GT –3′, reverse primer: 5′– ATG ATC CAG GCA GCG AAC AA –3′; *CHEK1*, forward primer: 5′– CTC CTC TAG CTC TGC TGC AT –3′, reverse primer: 5′– AAG TGA CTC GGG GCC TTT TT –3′; and β-actin, forward primer: 5′–TCC TCC CTG GAG AAG AGC TA–3′, reverse primer: 5′–CCA GAC AGC ACT GTG TTG GC–3′.

Western blotting

Cells were lysed with radioimmunoprecipitation assay (RIPA) buffer (Thermo, Waltham, MA, USA) containing proteinase inhibitors (Thermo). Cell lysates

were resolved by SDS-PAGE and transferred to polyvinylidene difluoride membranes (Bio-Rad Laboratories, Hercules, CA, USA) using Trans-Blot Turbo transfer system (Bio-Rad Laboratories). Membranes were blocked for 1 h at room temperature and incubated overnight at 4°C with primary antibodies, followed by incubation for 1 h at room temperature with horseradish peroxidase-conjugated secondary antibodies. Images were acquired with the C-Digit Blot Scanner (LI-COR, Lincoln, NE, USA). Anti-PAI1 (612024, BD Biosciences, San Jose, CA, USA, 1:1000), anti-YAP (sc-101199, Santa Cruz Biotechnology, Dallas, TX, USA, 1:200), anti-β-actin (sc-1615, Santa Cruz, 1:200), anti-α-tubulin (sc-5286, Santa Cruz, 1:200), anti-Lamin A (sc-20680, Santa Cruz, 1:200), anti-p53 (sc-6243, Santa Cruz, 1:200), anti-Rb (#9309, Cell Signaling Technology; CST, Danvers, MA, USA, 1:1000) were used as primary antibodies.

Apoptosis assay

Apoptosis assay was performed by Realtime-Glo Annexin V apoptosis and necrosis assay (JA1011, Promega, Madison, WI, USA) according to the manufacturer’s instruction. Briefly, cells were seeded at 10,000 cells/50 μl

media per well in 96-well plates and cultured for 24 h. Cells were then assigned to no treatment or 10 μM cisplatin (CDDP). The Luminescence and fluorescence were measured with a 96-well spectrophotometric plate reader (Synergy H1 microplate reader, BioTek, Santa Clara, CA, USA) at 0, 6, 12, and 24 hours after treatment.

Apoptosis was also assessed by Caspase-Glo 3/7 Assay (Promega) according to the manufacturer's instruction. Briefly, cells were seeded at 3,000 cells/100 μl media per well in 96-well plates and cultured for 24 h. Cells were then assigned to no treatment or 10 μM CDDP. The Luminescence was measured with a 96-well spectrophotometric plate reader (BioTek) at 24 hours after treatment. All experiments were performed in triplicate.

Cell viability assay

Cell viability assay was assessed by CellTiter-Glo Luminescent Cell Viability Assay (G7570, Promega, Madison, WI, USA) according to the manufacturer's protocol. Briefly, cells were seeded at 5000 cells/100 μl media per well in 96-well plates and cultured for 24 h. Cells were then assigned to no treatment or CDDP. After 24 h, CellTiter-Glo was added at 100 μl/well and cells were incubated for 10 min. The Luminescence was measured at 450 nm with a 96-

well spectrophotometric plate reader (BioTek). All experiments were performed in triplicate.

Scratch assay

Scratch assay was assessed by Cell Comb^{TM} Scratch Assay (17-10191, MilliporeSigma, Burlington, MA, USA) according to the manufacturer's protocol. Briefly, cells were seeded at $5x10^6$ cells in the plates and cultured for 24 h. After aspirating the media, cells were scratched with the teeth of the comb, then acquired the first image of scratch (0 h). Images were taken at 6 h, 12 h, and 24 h after incubation. Proportion of the wound healed was normalized using ImageJ (NIH) by the width at the 0 h. Five randomly chosen fields were quantitatively examined.

Clonogenic assay

Cells were seeded at 1000 cells/ml per well in 6-well plates in triplicate and cultured for 24 h. Cells were then treated with no treatment or CDDP at the final concentrations described above. After culture for 14 days, cells were washed, fixed in 6% glutaraldehyde (G0067, TCI, Tokyo, Japan) and stained with 0.5% crystal violet (548-62-9, Alfa Aesar, Haverhill, MA, USA) for 30 min. For quantification, 1 ml of 1% SDS (Invitrogen) was added to each plate for 30 min and then 200 µl solution was collected from each well and transferred to a 96-well plate. The absorbance at 595 nm was determined using a spectrophotometric plate reader (BioTek).

Human Apoptosis array

Human apoptosis array was performed by Human Apoptosis Array Kit (ARY 009, Promega) according to the manufacturer's instruction with 2.0 x 10^6 cells in 10 cm dish and cultured for 24 h. Cells were then assigned to no treatment or 10 μM CDDP. After 24 hours treatment, cells were rinsed with PBS and lysis buffer was added. Cell lysates were centrifuged at 14,000 x g for 5 minutes at 2-8 °C. Array membranes were incubated in blocking buffer for 1 hour. 300 μg / 250 μl of cell lysates were applied on array membranes and incubated overnight at 4 °C on a rocking platform shaker. After washing each membrane 3 times for 10 minutes, membranes were incubated in detection antibody cocktail for 1 hour. Then, membranes were washed 3 times and incubated in streptavidin-HRP for 30 minutes. Chemi Reagent Mix was added and allowed to incubated for 1 minute. Images and quantifications were acquired with the C-Digit Blot Scanner (LI-COR, Lincoln, NE, USA) and Image Studio (LI-COR), respectively.

Immunocytochemistry (ICC)

BrdU immunocytochemistry was performed by using BrdU immunohistochemistry Kit (ab125306, Abcam, Cambridge, UK) according to the

manufacturer's instruction. Briefly, cells were seeded in Poly-Lysine coated 8-well chamber slide plates (Corning, NY, USA) at 10,000 cells / 100 µl per well and cultured for 24 h. Existing culture medium was removed from the cells and replaced with 10 µM BrdU (ab142567, Abcam) labeling solution and incubated for 2 hours. Cells were fixed in 70% ethanol at room temperature for 30 min. After blocking endogenous peroxidase activity using in 3% H_2O_2 in PBS for 10 min, 2 drops of denaturing solution were added to each well and incubated at room temperature for 30 min. After removing the denaturing solution, cells were blocked with 2 drops of blocking buffer per well for 10 min at room temperature, then incubated with primary antibody at room temperature for 60 min. Two drops of streptavidin-HRP conjugate was added to each well after the primary antibody solution had been removed and the slides had been washed twice in PBS for two minutes. Next, 50 µL of DAB Solution was added to each well and incubated at room temperature for 10 min. After washing 2 times for 2 min, 1 drop of hematoxylin was added and allowed to incubate at room temperature for 3 min, then incubated for 1 min in PBS until the staining color color turned blue. After removing the chamber, slides were dehydrated with ethanol and xylene, and 1-2 drops of mounting media was added along with a coverslip. Five

randomly chosen fields were quantitatively examined with ImageJ (National Institutes of Health, Bethesda, MD, USA) to determine the BrdU-positive cell area.

Cellular fractionation

Cellular fractionation was performed as previously described. Briefly, 8×10^6 cells were plated for high concentration (HC), or 1×10^6 cells for low concentration (LC) in 10 cm dish and incubated for 24 hours. Cells were trypsinized and collected by centrifugation at 400 × g for 5 min, then washed with ice-cold DPBS. The cells were solubilized in ice-cold 0.1% Nonidet P (NP) 40 (Calbiochem, San Diego, CA, USA)-PBS containing protease/phosphatase inhibitor (Thermo). Cytosolic and nuclear fractions were separated by centrifugation at 10,000 × g for 3 min at 4 °C. The nuclear pellet was washed once in 0.1% NP40-PBS and centrifuged at 10,000 × g for 3 min at 4 °C. For western blotting tests, the nuclear pellet was resuspended in RIPA buffer. Western blotting was performed with 30 μg of protein from each fraction.

Small interfering RNA (siRNA) transfection

Cells were seeded at 5 x 10^3 cells per well of a 96-well plate in 100 µl of RPMI for 5637 or low glucose DMEM for UROtsa containing 10% FBS, 1% Penicillin-Streptomycin and incubated at 37°C and 5% CO_2 for 24 h. The predesigned siRNA *CLSPN1*#1 and scrambled negative control (SCR) siRNA (Qiagen) were purchased from Qiagen. Cells were transfected with siRNA at 5 nM for UROtsa or 2 nM for 5637 using INTERFERin transfection reagent (Polyplus) in OptiMEM GlutaMAX serum-free media (Gibco). After incubation for 48 h, cells were harvested and subjected to further analysis.
